# The role of communities in sustainable land and forest management: The case of Nyanga, Zvimba and Guruve districts of Zimbabwe

**DOI:** 10.4102/jamba.v8i3.281

**Published:** 2016-05-11

**Authors:** Diego Matsvange, Ruvimbo Sagonda, Munyaradzi Kaundikiza

**Affiliations:** 1 Environmental Education Association of Southern Africa, Gweru, Zimbabwe

## Abstract

Forest benefit analysis is vital in ensuring sustainable community-based natural resources management. Forest depletion and degradation are key issues in rural Zimbabwe and strategies to enhance sustainable forest management are continually sought. This study was carried out to assess the impact of forests on communities from Nyanga, Guruve and Zvimba districts of Zimbabwe. It is based on a Big Lottery Fund project implemented by Progressio-UK and Environment Africa. It focuses on identifying replicable community forest and land management strategies and the level of benefits accruing to the community. Analysis of change was based on the Income and Food Security and Forest benefits, which also constitutes the tools used during the research. The study confirms the high rate of deforestation and the increased realisation by communities to initiate practical measures aimed at protecting and sustaining forest and land resources from which they derive economic and social benefits. The results highlight the value of community structures (Farmer Field Schools and Environmental Action Groups) as conduits for natural resource management. The interconnectivity among forests, agricultural systems and the integral role of people are recognised as key to climate change adaptation.

## Introduction

Progressio-UK and Environment Africa (EA) are implementing a project entitled ‘Conserving our Land and Producing Food’ in Guruve, Zvimba and Nyanga districts of Zimbabwe. It was anticipated that at the end of the project, there will be an increase in household income and food security of poor and marginalised communities through agro-ecology and sustainable, equitable farming approaches and access to markets. An increase in sustainable management of forest, land and water resources is expected for the benefit of the most disadvantaged households. Local communities are expected to be able to engage with local and national governments to ensure better management and use of natural resources.

The project makes use of community-based groupings, namely the Environmental Action Groups (EAGs) and Farmer Field Schools (FFS), which have gone through an intensive capacity-building in forest management.

### Background to study

This study is a culmination of a series of observations, monitoring and evaluation and results of Regular Impact for Capacity Assessment (RICA) surveys. It largely focuses on the work of EAGs and FFS. It examines the current scenario against two major RICA indicators of forest benefits and income or food security expected to be accelerated by environmental education (EE) and sustainable development.

## Materials and methods

The methodology adopted for this evaluation was designed to capture both qualitative and quantitative information. The methodological mix comprises the use of weighted semi-structured questionnaires for individual households, focus group discussions (FGDs) and observations.

### Sampling design

The three wards in each of the three districts were purposively selected while randomised systematic sampling was used to select persons for individual interviews. Adult Big Lottery Fund beneficiaries constituted the largest proportion of FGDs. Consideration was also given to local opinion leaders and non-project beneficiaries to capture both negative and positive perceptions about the project.

### Individual interviews and focus group discussions

A total of 214 individual interviews from systematically selected respondents were conducted from the three operational districts. A semi-structured questionnaire was also used to capture information from 9 FGDs comprising a total of 115 beneficiaries.

#### The analytical framework

**FIGURE 1:**
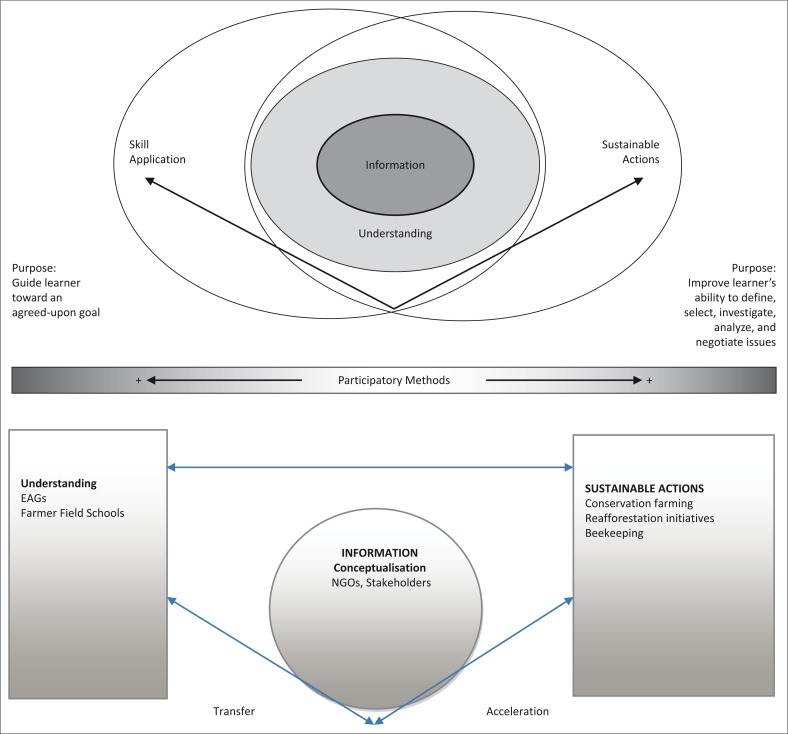
The environmental education strategies framework.

In interpreting the role of EAGs and FFS, the Environmental Education Strategies Framework (EESF) was used to assess the contribution that project activities have made to sustaining environmental management and sustainable actions. This model systematically defines four categories based on given objectives, including the conveyance of information, building understanding, improving skills and enabling sustainable actions (Figure 1). It is our understanding that the framework embraces participatory methods to ensure effective learning. The more participatory the educator is, and the extent to which the educator consults with, engages and collaborates with the audience, the greater the likelihood of achieving the educator’s objectives. The EESF is supported by RICA, which complements the framework by measuring the impact of EE through changes in the quality of life reported by a sample of poor and marginalised people in the project areas. It also measures the level of sustainable benefits reported as a result of sustainable practices.

#### Application of the Environmental Education Strategies Framework

In this modified model of the EESF, it is assumed that conceptualisation takes place within the stakeholder sphere, where a number of actors tend to congregate. Of significance are government departments, including Department of Agriculture and Technical Extension Services (AGRITEX), Environmental Management Agency (EMA) and Forestry Commission (FC). Equally important are nongovernmental organisations such as EA and Progressio. These actors bring in information and target individuals in FFS and EAGs, with the hope that providing information will change attitudes and behaviour. According to Monroe *et al*. (2013), the targets for most EE programmes are individuals because they have the potential to change their behaviour. Therefore, knowledge is transferred to individuals in FFS and EAGs who in turn disseminate the same to the larger community. This model takes advantage of the social capital, which exists in communities, taking into cognizance that social capital is a vital ingredient that strengthens and enhances a community (Monroe *et al*. 2013). It is necessary in fostering networks for collective action. Such networks, built through trust, determine the ability of a group to solve problems successfully (Monroe *et al*. 2013). Therefore, in this case, relationships created through the EAGs and FFS drive the communities to apply their knowledge, while at the same time addressing key environmental challenges.

## Main findings

### Forest benefits

#### Training received for sustainable forest exploitation

**FIGURE 2:**
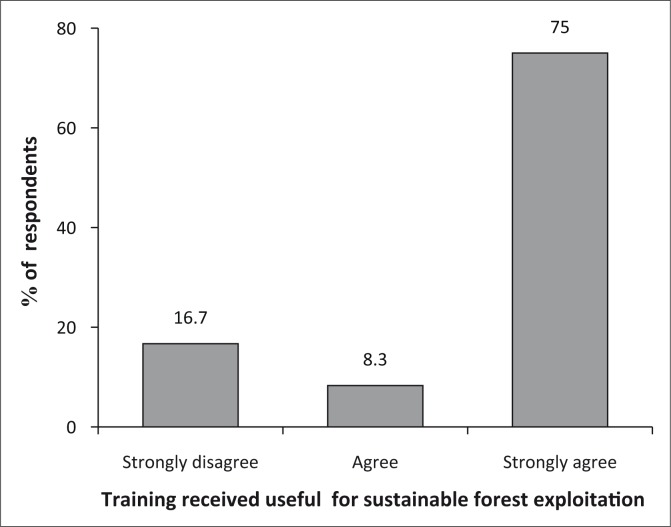
Training received for sustainable forest exploitation.

In an attempt to cultivate the culture of sustainable forestry management, it was encouraging to realise that 75% of the community confirmed having received useful training and awareness on sustainable forest management. The training was received through the FFS and EAG model (see Figure 2).

#### Training enabling an improvement in forest cover

**FIGURE 3:**
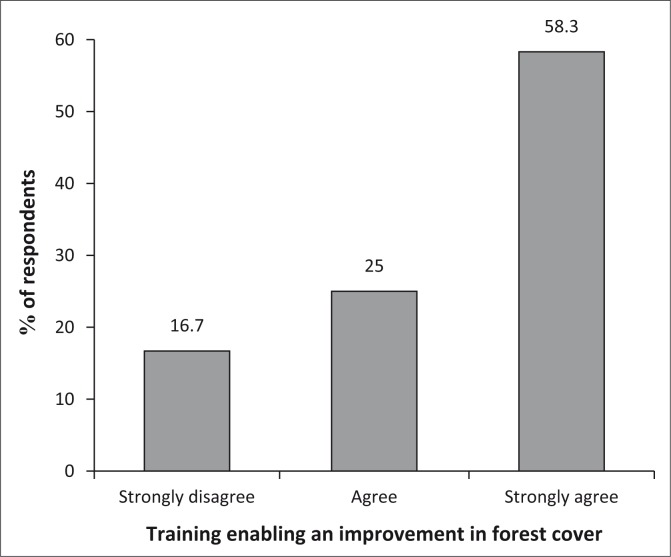
Training enabling an improvement in forest cover.

The series of trainings and awareness sessions carried out have seen a significant improvement in forest density. Figure 3 demonstrates that 58% of the population agree to this, and further in-depth interview revealed that following the trainings, some community members have organised tree planting activities on selected parts of their forest.

#### Community Initiatives giving locals greater control over forests

**FIGURE 4:**
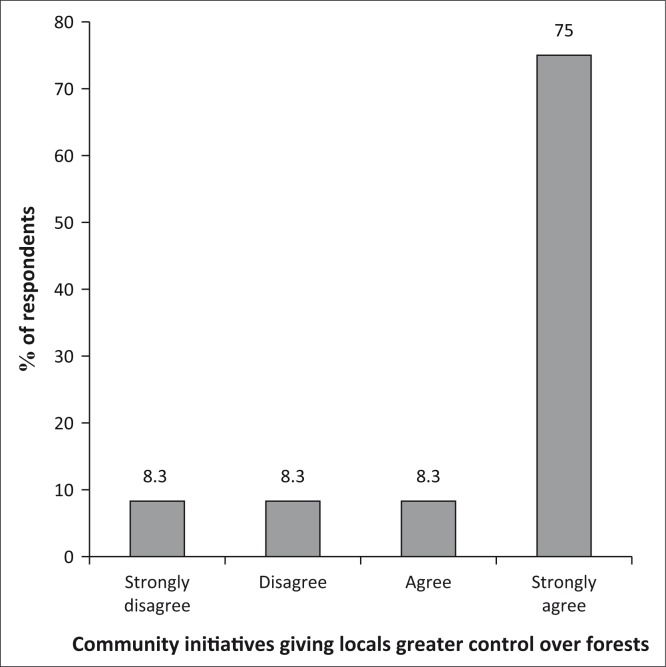
Community initiatives giving locals greater control over forests.

Of the population, 75% believe that the community now has greater control over their forests as a result of locally driven initiatives as shown in Figure 4. Such initiatives include tree planting, use of alternative sources of energy (still very low though), the building of fire barriers and bee keeping.

#### Perceptions on changes in forest diversity in the past 20 years

**FIGURE 5:**
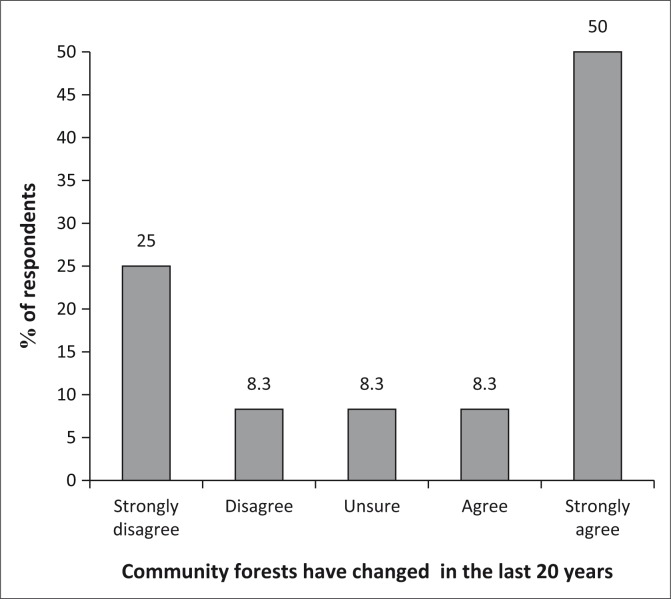
Changes in community forests in the past 20 years.

As shown in Figure 5, it was disheartening to note that over the past 20 years, forestry diversity has been lost as confirmed by close to 70% of the population. A number of indigenous fruit trees in particular have become extinct, while others have been decimated to critically low levels.

#### Perception on forest size changes in the past 20 years

**FIGURE 6:**
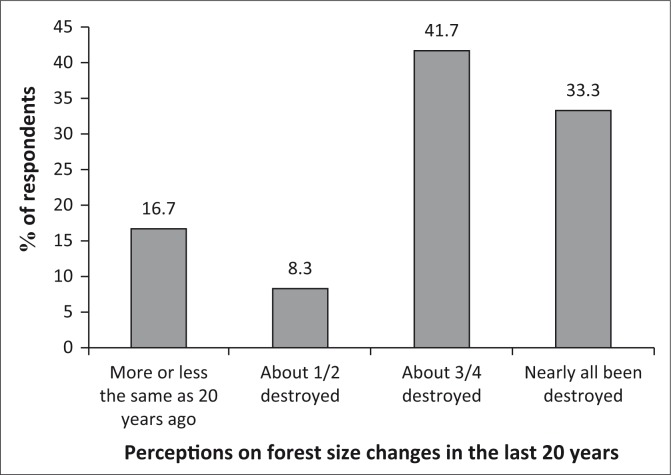
Changes in forest size in 20 years.

Communities in the three districts concur on the fact there has been massive destruction of forests size since 20 years ago. In all, 42% perceive that about 75% of the forests have been destroyed, while 33% noted that all has been destroyed. This is illustrated in Figure 6. Every year, at least 70 000 ha of forests are destroyed in Zimbabwe (Gwaze & Marunda 2014).

#### Deforestation problems

**FIGURE 7:**
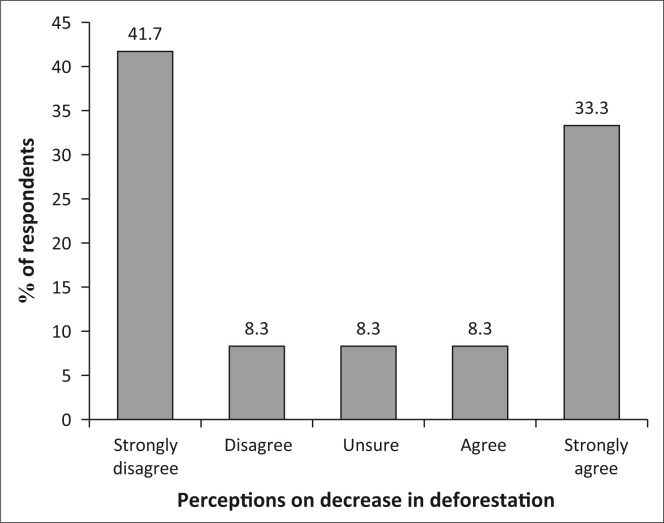
Decrease in problems related to deforestation.

Because of the ongoing activities in agrarian reform, access to community forests in some districts such as Nyanga was heavily affected because a significant amount of forestry land was turned into semi-commercial farming areas. This was not common in other districts such as Guruve. Therefore, while 33% of the population strongly agree to a decrease in the loss of forests for commercial purposes and other uses, 42% strongly disagree as illustrated in Figure 7.

#### Access to forests because of preservation initiatives

**FIGURE 8:**
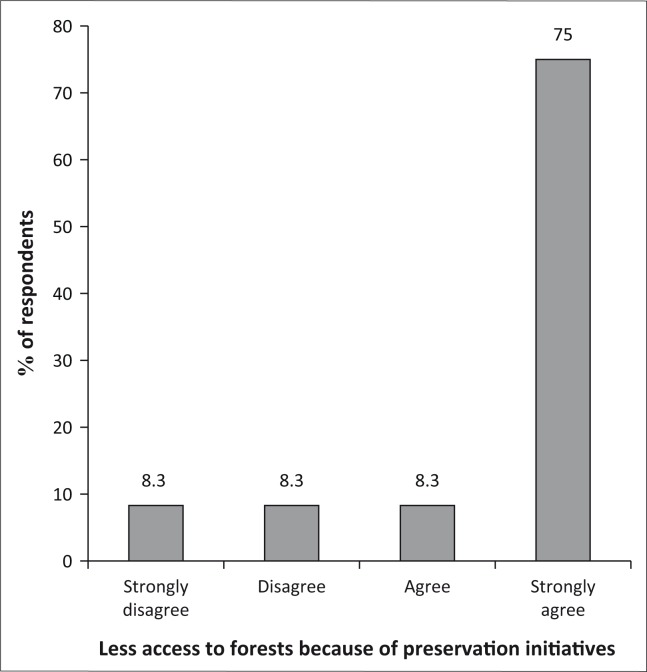
Access to forests because of preservation initiatives.

It was evident that the community (75%) strongly agrees that there is now limited access to forests because of initiatives put in place to preserve them as shown in Figure 8. This in a way is a positive development because it might imply better forest preservation, though the policies implemented at the community level must be developed to ensure sustainable exploitation of forestry products.

### Income and food security

#### Quality of life

**FIGURE 9:**
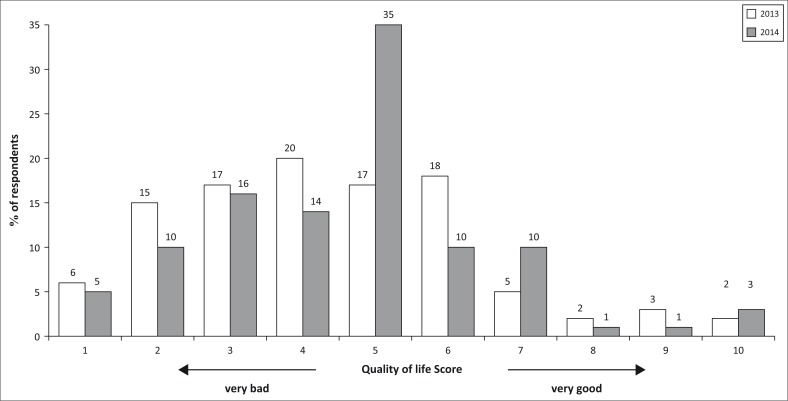
Quality of life over the past 2 years (2013–2014).

This section was aimed at examining the quality of life (see Figure 9) as a result of the effects of EE. Using the quality of life continuum/scale it was evident that there was a positive shift in the quality of life in the current year (2014) as compared to previous years. Of the beneficiaries, 75% had a quality of life ranging from 1 to 5 during the year 2013 as compared to 80% in 2014. However, in 2014 there is a cumulative total of 63% of the population as compared to 50% in 2013 with a quality of life ranging between 5 and 10. A quality of life ranging from 5 to 10 indicates positive outcomes from natural resource exploitation.

Of the 25% enjoying a better quality of life (6–10) in 2014, 80% of them have a quality of life between 6 and 7 representing 20% of the entire population. During the previous year (2013), the (6–10) quality of life was enjoyed by 28% of the population. The margin of change in the quality of life for the 2 years under comparison is small though it is projected to increase as people continue to sustainably exploit their forests for livelihoods benefits.

#### Changes in sources of income

**FIGURE 10:**
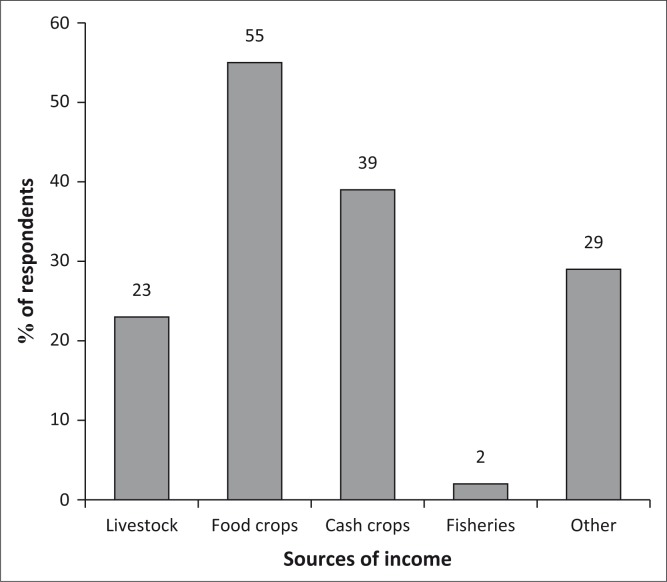
Sources of income.

It was evident that the households have more than one source of income as shown Figure 10. The sale of food crops was identified as the major source of income by 55% of the population, followed by cash crops (29%), while 23% derive income from livestock and 29% have other sources of income.

The diversification of income sources and particularly the increase in crop sales was also testified by 29% of the population as having increased the level of income as compared to 2 years ago.

#### Access to food

**FIGURE 11:**
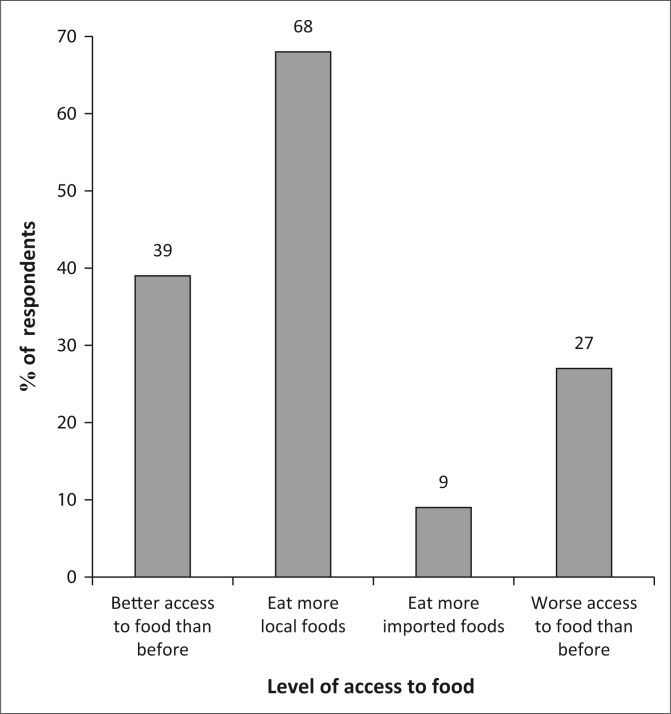
Access to food.

Of the respondents, 68% rely on locally produced foods and 9% rely on imported foods as illustrated in Figure 11. As a result, this is evidence of increasing food productivity. These are more likely to be active in several income-generating activities described above. Significantly, 29% of the population currently have access to food than before, despite the poor yields in the current year.

#### Farming practices

**FIGURE 12:**
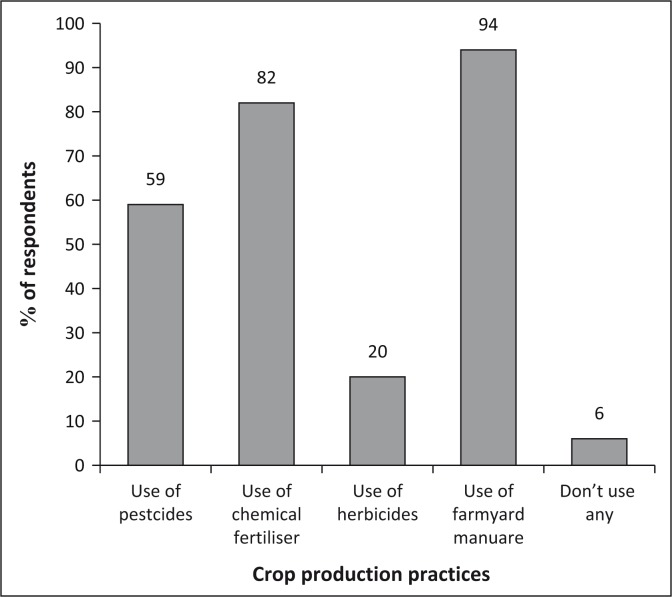
Crop production practices.

The use of organic materials, such as manure and compost, is the most common crop management practice in the three districts. This is being done by 94% of the population, while a subset (82%) of these also uses fertilisers. The use of pesticides is mainly common in the gardens, being used by 59%. Herbicide use is the least (20%) common, although significant (see Figure 12).

**FIGURE 13:**
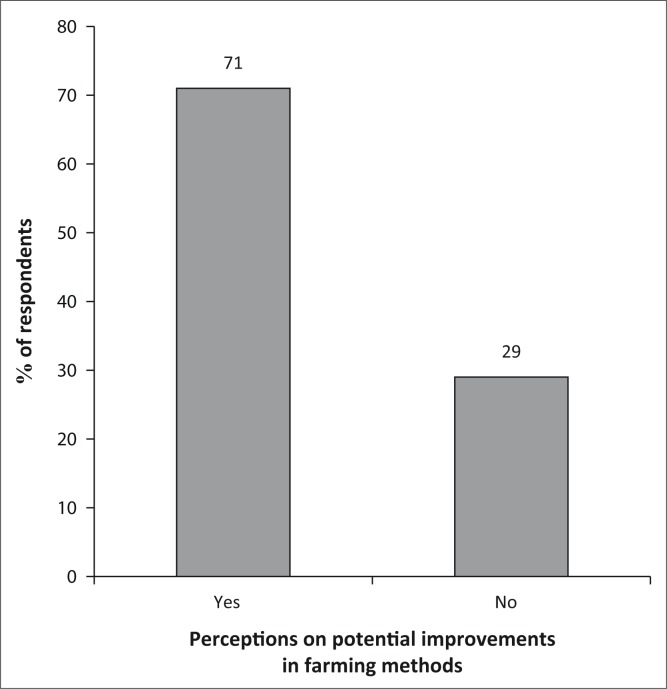
Improvements in farming methods.

Closely linked to the improvements in farming methods (Figure 13) is the fact that a significant proportion of the population (60%) recorded an increase in the quantity of food produced. The quality of produce was also noted by 48% of the population as a result of new farming methods. Of interest is the small fraction of the population (22%) who felt that the farming methods have increased the time spent in the production process. Justifiably, 78% of the population did not experience the lengthening of time in the production process.

#### Effects of new farming methods on land quality

**FIGURE 14:**
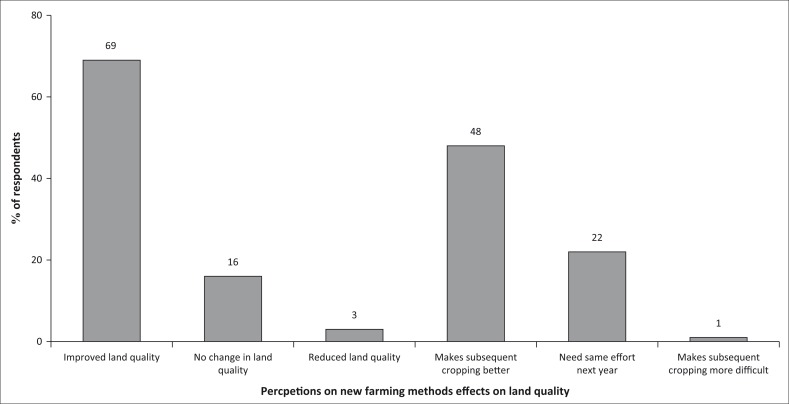
Effects of new farming methods on land quality.

Figure 14 shows that the new farming methods being promoted by the project have a significant effect on land quality and subsequent cropping. Of the respondents, 69% agreed that the methods have improved the quality of their land while 48% testify that the farming methods have made subsequent cropping much easier. However, 16% argued that there is no change in land quality as a result of the promoted farming methods.

## Discussion

### The role of education in sustainable forest management and exploitation

Environmental adult education seeks to empower the individuals and encourages them to be activists in order to fight the causes of environmental problems. The EAG approach applied in the EESF model is an institution for environmental activism. Environmental adult education strives to involve learners from all positions in the society and recognises the role of all stakeholders (Haugen 2010). Environmental adult education emphasises on examination of environmental problems, (UNESCO 1999). Its weakness is that it tends to focus on expert knowledge at the expense of traditional knowledge systems, and EE is less developed in the adult education form than it is in the formal education system (UNESCO 1999). The EESF model proposed in this research takes the same approach, but considers both indigenous knowledge systems and expert knowledge, which is provided by extension workers.

According to Anderson and Strecker (2012), education can enable individuals to make informed decisions and to take action towards sustainable development. According to the same author, sustainable development can be achieved through a dynamic process of adaptation, learning and action, which has been expressed in this research through the FFS approach. Such educational interventions can be more successful when they address local, tangible and actionable environmental management practices which can be addressed by individuals (Anderson & Strecker 2012).

After all the transformations implied in this discussion have occurred, what matters most is for communities to be able to improve their livelihood systems from the forest resources (Matose 2008). As in the Nyanga case, EAGs have been motivated to initiate beekeeping activities, not only for income generation but also in order to manage their indigenous forests. Where beehives have been mounted, incidences of rampant cutting down of trees have become fewer particularly in Nyanga. The EAGs have also been motivated to construct fireguards around their indigenous as well as individual woodlots, to protect the beehives mounted in such areas. As a result, there have also been fewer incidences of forest fires in their forests. In addition to managing their forests, the communities have also been able to secure a source of income. The result is innovation, which is described as being driven by attitudes and skills (Schmitz, Stinson & James 2010).

### Changes in forest diversity and size

The results of this research have seen community perceiving a decline in forest diversity and size during the past 20 years. This concurs with national and global trends, where forests are declining because of several human activities, such as increase in deforestation and increase in demand for firewood, especially in Zimbabwe with increase in power cuts. Bawa and Seidler (1998) indicate an increase in mean annual losses from 6% between 1970 and 1980 to 8% between 1980 and 1990. FAO statistics also indicate a decline in the extent of forests and other wooded land between 1990 and 2005. In 1990, the statistics show that approximately 27 671 000 ha were under forest and other wooded land and this figure declined to 17 540 000 ha in 2005 in Zimbabwe (UNCSD 2008). A global deforestation rate of 10 million hectares per year has been recorded (Rametsteiner & Simula 2013). Removal of about 3% of trees in a forest reduces forest cover by approximately 50% (Bawa & Seidler 1998). According to a research by FAO (2013), Zimbabwe is among the 10 countries in the world that has lost more forest area between 1990 and 2010. Deforestation in Zimbabwe is expounded by agricultural expansion and by an increase in demand for firewood (UNSCD 2008).

The drivers of decline in forest diversity and size include an increase in demand for construction timber and more recently an increase in demand for firewood for curing tobacco. This has been necessitated by high electricity costs and lack of financial resources to purchase alternative sources of fuel such as coal and biogas (Marufu 2012). Therefore, community perspectives can be confirmed by documenting global and national trends with regard to deforestation.

### Community involvement in forest management

Isager, Theilade and Thomson (2002) argue that successful conservation of forest resources depends on the full participation of communities. He also argues that the best formula for doing that is when communities work together with other stakeholders (Isager *et al*. 2002). The RICA results indicated that the majority of the community agrees that there is a positive shift towards community participation in forest management and protection. Through education, indabas and discussion sessions with the participating members of the EAGs, members have realised that the current pressures and problems bedevilling them are a result of their agricultural and livelihoods activities. The farmer groups were trained in forest-based enterprises such as beekeeping. As a result, they have realised that forests are an integral part of the beekeeping enterprise. Therefore, the farmers now jealously guard and work to improve on the forest health and vitality, species diversity, the productive functions and the general forest protection, especially from the vice of veldt fires and deforestation tendencies by human activities in the area. This is the most appropriate way of involving communities in the sustainable management of forests as their efforts are directly rewarded by way of honey harvests, which generate family income. Once the farmers enjoy the benefits of the forests, they may not be forced into protecting them. It then becomes automatic.

### Consideration of community needs in forest management

Worldwide, approximately 60 million people depend entirely on trees and forests for a living, while 350 million depend on trees for subsistence and income. The research indicates that majority of communities agree that their needs are being considered through the forest management initiatives. Matiku, Caleb and Callistus (2013) value some of the forest products considered as needs by local communities. These include firewood, building materials, non-timber forest products (such as honey, mushrooms and butterfly pupae), fruits and herbal medicines (Matiku *et al*. 2013; CIFOR 2014). Trees also make an essential contribution to food and nutrition as well as income (which is also needed in order to secure food) (Matiku *et* *al*. 2013). Some authors also describe the non-cash forest functions, which communities depend on, which include provision of forestry resources for agriculture purposes and climate change mitigation (Spittlehouse & Stewart 2003). Forests also provide services such as soil and water protection, maintenance of soil quality, regulation of local climate, provision of habitats for useful agricultural pests and storing biodiversity, services which are crucial in agriculture (CIFOR 2014). A significant number of people in the world rely on agroforestry systems for subsistence (Sam & Shepherd 2011).

In this regard, communities have started appreciating the interventions because some have started paying dividends with some early planted exotic trees now being harvested for various uses by the villagers. However, Isager *et al*. (2002) argue that the interests of people in forests are usually more than financial. The importance of planting and taking care of the trees has shifted from being a verbal educational programme to some reality that they are witnessing and living. With more benefits being accrued form forest management, less effort will be needed to persuade and encourage the communities to plant and manage trees and forests.

### Control of forests and use of natural products

The lack of empowerment of local communities in decisions over forest resources as one of the key natural resource management issues has contributed to deforestation in Zimbabwe (Marufu 2012). In addition, some of the existing legal frameworks do not clearly give local communities control over their natural resources, but rather they specify how communities should exploit their natural resources. Community rights of access and use were not prioritised because of low population densities (Matiku *et al*. 2013). With time, as the population densities increased, permit-based system was introduced, which undermined customary management systems. The failure of such approaches prompted the introduction of co-management in which the role of communities began to be appreciated (Matiku *et al*. 2013).

However, the EAG approach highlighted earlier in this report enables locals to establish an institution which work not only as an environmental watchdog but also as one in which communities are equipped to lobby authorities for better management of natural resources including forests (Fisher, Prabhu & Mcdougall 2007). It also enables communities to take action to manage their resources, as what has been highlighted in this research, through activities such as tree planting, beekeeping and establishment of community woodlots among others. In applying the EAG concept, the community members are trained to engage their local leaders, as well as other relevant stakeholders. It creates a platform for exchange of information between government, stakeholders and local people (Matose 2008). As illustrated earlier in the EES Framework, communities do not work in a vacuum, but with support from the local leaders and other stakeholders. In some instances, for example, in Zvimba, the kraal heads are also members of EAGs. In such cases, they become key and strengthen the work of the EAGs.

In a bid to advocate for the local leaders’ buy in into the issues to do with sustainable management of natural resources, there was a programme that trained (educated) the local leaders on their roles in national resource management. The trainings emphasised the roles of leaders as outlined in sections and subsections of the *Forestry Act of 1998*, the *EMA Act* and *Rural District Council (RDC) Act*. The trainings would remind the leaders of their mandate as well as the powers vested in them by these acts. Such trainings have benefited the leaders by enlightening them on how they should coordinate issues to do with the environmental management and also helped to strengthen their relationship with local communities.

Institutions such as the EAGs enable collaboration, which improves chances of learning from one another and can lead to better decision-making (Fisher *et al*. 2007). This is possible because different stakeholders bring in different knowledge, experiences, perspectives, values and capacities (Fisher *et al*. 2007). They also create an opportunity for locals to participate in the modification of local rules for management of resources as was the case in the development of bylaws in the Guruve district (Matose 2008). However, the EAGs need to be supported by financial and institutional resources to empower communities not only to be labourers but also to be able to make their own decisions (Matiku *et al*. 2013). Most importantly, they need to be equipped with education and rights to increase their accountability at the local level (Shyamsundar & Ghate 2011).

This research has highlighted responses from communities which indicated that half have been able to use natural resources from their forest, while half disagree. Such responses can both be accepted. In Nyanga, where communities have been able to use beekeeping as a means to manage their indigenous forests, people have already been able to benefit from natural products such as honey and related products. Beekeeping is a short-term project whose benefits can be reaped within a year after the start of the project. In some cases, honey production is possible twice in a year where there is adequate water supply, forage and favourable weather conditions. In other districts, the approach is more reactive. There is a need to engage in afforestation projects to restore forest cover, the benefits may therefore be long term.

### Use of forests in a sustainable way

There was strong contention on whether community members have demonstrated sustainable management of forests during the 2 years. Sustainable utilisation of forest resources involves harvesting the resources in a way that will allow them to regenerate naturally before the next harvest (Bawa & Seidler 1998). There are some interventions in some communities that are working directly with products from the forests and other natural resources, for example Guruve and Zvimba are involved in conservation farming and alternative energy technologies. Nyanga wards are engaged in beekeeping as their main activity in that district. Other activities include tree planting, particularly focusing on fast-growing exotic species such as the gum tree. Gum trees take a short period of time to mature such that harvesting takes place after a minimum of 8 years. The eucalyptus trees have the advantage of fast re-growth after each harvest, making them the best option under the current environment. That also helps to reduce the pressure on indigenous forests and guarantees the continued supply of forest products.

Biogas plants are one alternative source of energy households can use instead of firewood. Use of alternative energy sources means that households do not need to travel long distances in search of firewood. When households reduce firewood consumption, the pressure on forests also reduces. Funds are made available (as interest-free loans) to community members for them to construct biogas plants at their homesteads and repay the principal loan over a period to help in increasing the adoption of the technology by community members. So as the call to reduce cutting down of trees grows, an alternative which the communities can adopt has been made available.

Trees and forests have cultural, spiritual, recreational and aesthetic values, which are often undervalued by technocrats (Isager *et al*. 2002). The interests of local communities on trees and forests are more than just financial (Isager *et al*. 2002). However, communities consider trees and forest to be significant for the purpose of such benefits in their livelihoods and thus apply a cultural norm to protect them. Applying cultural norms helps in protecting some tree species that are regarded as more special to these communities. Such norms are applied either to specific trees or to specific forest areas. This has resulted in these communities preserving the forests or same trees for their benefits. That contributes towards the preservation of the biodiversity in the community environs.

Income generation through beekeeping provides an incentive to conserve the forests. Villagers are comfortable with activities that give direct benefits. Perpetuation of the benefits also perpetuates the practice that brings about the benefits. Villagers are motivated to continue taking care of their forest because of the associated benefits. This kind of attitude and commitment is quite positive and will in the long term pay dividends if the communities remain resolute and steadfast about the environmental issues (Mogaka *et al*. 2001).

### Decrease in problems related to deforestation

This discussion establishes efforts by communities to reflect on typical changes relating to forest protection and management of other natural resources. The older generations who had the opportunity to witness both situations in the 1950s – 1960s and the current situation have a clear picture for comparing change. Communities have now come to realise that their current problems of shortages (of forest products) are as a result of their activities.

EE programmes for reafforestation, conservation and sustainable utilisation of natural resources have been key in accelerating sustainable forest resources utilisation and management.

The RICA survey has established that communities strongly disagree with the notion that there has been a reduction in deforestation in their surroundings. This is because the environs are still without big trees, which is an indication that the problem of deforestation is still persisting to some extent, especially in the distant forests. The observation is true because the problem is still ongoing, although it is at a reduced scale in comparative terms. The continuation could be attributed to the non-availability of better alternative options available to the families. The same communities had received EE and understood, but because they are faced with pressing life requirements especially of household energy, they are left with no option but to resort to the old way of doing things. The education programmes are prescribing long-term solutions to the current problems in the communities, which are acceptable, but the same communities have problems that require immediate solutions, such as the biogas and wood-saving stoves as alternative sources of energy.

Communities confirmed that there has been an increase in forest cover. They attribute this change to the training they have received. Indeed, as a result of community activities, the potential for increasing forest cover is higher. The research confirms that at least 40 woodlots were established in Nyanga, Guruve and Zvimba, each covering on average 0.4 ha per community group. This brings the total to 16 ha of new woodlots. In addition, there has been an increase in the management of indigenous forests, resulting in fewer veldt fires and reduced felling of trees. Management of forests by the communities has potential to maintain forest cover as well as to allow regeneration of trees within managed areas.

Because of the EE and awareness programmes, some communities in the three districts have taken the initiatives seriously, resulting in the 40 woodlots established. Voluntary efforts are being done by some members, especially in Zvimba where the EAGs are organising themselves for collective protection and sustainable utilisation of the wetlands (for gardening and livestock watering). The motivating factor is that the communities have come to realise the social, ecological and economic relevance of these resources to their livelihoods and further motivation is not required to take care of them.

The well-established EAGs are now making deliberate and conscious efforts to preserve what is left of their forests in the environs, thus allowing for the regeneration of the forests (Bawa & Seidler 1998). Efforts are also focused on the reduction of the effects of veldt fires by fighting fire outbreaks through fire-fighting committees and also reducing the incidences where fire is deliberately used for agricultural land preparations. In Zvimba District wards, there was a marked decrease (about 58%) in the hectares of veldt that was destroyed by fires in the past 2 years as compared to 5 years ago.

### Income and food security benefits

#### Changes in sources of income

The diversification of income sources and particularly the increase in crop sales was also testified by 29% of the population as having increased the level of income as compared to 2 years ago. Grabowski (2011) sited the following as some important non-monetary benefits brought about by conservation farming:

- facilitates early planting
- reduced labour bottleneck at planting time
- increased soil water availability: reduced evaporation and run-off and increased infiltration
- temperature regulation
- educed erosion
- increased organic matter accumulation
- improved soil structure, aeration, water penetration and microbial build-up
- increased nitrogen mineralisation.

Matiku *et al*. (2013) identified several benefits of the forest to nearby communities, such as firewood extraction taking $51 of the household income, non-timber forest products ($39), building materials ($50), herbal medicines ($9), forest-related employment contributing $333 in income to 4% of the households, drinking water to 2% and livestock grazing to 0.3% of the study sample.

### Access to food

The improved access to food by households can be attributed to the EE programmes introduced in the districts and wards in particular by EA training programmes during the course of the implementation of interventions. The EE programmes in agriculture articulated a number of concepts that include conservation farming and agro-ecology laying out the underlying principles.

### Effects of new farming methods on land quality

The recognisable effects of new farming methods promoted by EE programmes include the following:

- Rebuilding or replenishing of soil fertility that has been depleted over generations of farming activities. Agroforestry practices of using leguminous trees and shrubs in Conservation agriculture (CA) rotations help in increasing nitrogen from the nitrogen-fixing bacteria found in the root nodules.
- Improvement in soil structure, aeration, water penetration and retention, resistance to erosion and nutrient retention ability. Rotations with non-legumes can result in relative yield increases, possibly because of increased.
- Water infiltration caused by higher levels of soil organisms such as earthworms.
- Important microbial build-up promoted by use of organic manure and reduction in chemical fertiliser usage.
- Reduced pollution of ground and surface water reservoirs.
- Reduction in veldt fires allowing a build-up of moribund essential in nutrient cycle systems
- Allowing regeneration of natural forests.

## Conclusion

Indeed, there has been a decline in forest cover in Zimbabwe over the past 20 years, because of an increase in deforestation. With a business-as-usual scenario, the rate of deforestation could reach alarming rates because of rising trends such as increase in the veldt and forest fires, increase in demand for firewood for curing tobacco in addition to the conventional causes of deforestation.

EE is an important precursor to sustainable forest management. Not only does it facilitate access to forest resources but also it gives communities knowledge and power to manage the resources sustainably. The research has highlighted the importance of EE to improving the livelihoods of forest-dependent communities.

The FFS approach is not only a means to enhance the livelihoods of rural households but also a vital tool for addressing environmental challenges such as deforestation, through empowering communities to take full custodianship of their natural resources.

The FFS/EAG approach also is vital in bringing together different stakeholders to train organised community groups and covering specific areas of expertise. Resources can be pooled together, thus reducing costs of training or building the capacity of the FFS members.

Giving communities control over resources within their local areas, which could otherwise be regarded as a common property, gives them the responsibility to manage the use of these resources and rehabilitate the environmentally degraded areas. Communities are motivated to come up with local initiatives to improve their environment. This could go a long way in relieving the government, which is usually constrained in terms of funds or human resources to cover large areas.

Most of the communities have come to realise that most environmental challenges faced today are man-made and it is their responsibility to reverse the situation and reclaim the lost natural status.

Communities receiving EE are usually faced with a dilemma of understanding the need to conserve the natural resources and allowing them to regenerate on one hand while on the other hand they need to use the resources for daily requirements.
